# Development of a rapid, low-cost protoplast transfection system for switchgrass (*Panicum virgatum* L.)

**DOI:** 10.1007/s00299-015-1913-7

**Published:** 2015-12-21

**Authors:** Kellie P. Burris, Elizabeth M. Dlugosz, A. Grace Collins, C. Neal Stewart, Scott C. Lenaghan

**Affiliations:** Department of Plant Sciences, University of Tennessee, Knoxville, TN 37996 USA; Center for Renewable Carbon, University of Tennessee, Knoxville, TN 37996 USA; Department of Mechanical, Aerospace, and Biomedical Engineering, University of Tennessee, Knoxville, TN 37996 USA

**Keywords:** Switchgrass, Protoplasts, Transformation, Enzymatic digestion

## Abstract

*****Key message***:**

**A switchgrass protoplast system was developed, achieving a cost reduction of ~1000-fold, a threefold increase in transformation efficiency, and a fourfold reduction in required DNA quantity compared to previous methods.**

**Abstract:**

In recent years, there has been a resurgence in the use of protoplast systems for rapid screening of gene silencing and genome-editing targets for siRNA, miRNA, and CRISPR technologies. In the case of switchgrass (*Panicum virgatum* L.), to achieve economic feasibility for biofuel production, it is necessary to develop plants with decreased cell wall recalcitrance to reduce processing costs. To achieve this goal, transgenic plants have been generated with altered cell wall chemistry; however, with limited success owing to the complexity of cell walls. Because of the considerable cost, time, and effort required to screen transgenic plants, a protoplast system that can provide data at an early stage has potential to eliminate low performing candidate genes/targets prior to the creation of transgenic plants. Despite the advantages of protoplast systems, protoplast isolation in switchgrass has proven costly, requiring expensive lab-grade enzymes and high DNA quantities. In this paper, we describe a low-cost protoplast isolation system using a mesophyll culture approach and a cell suspension culture. Results from this work show a cost reduction of ~1000-fold compared to previous methods of protoplast isolation in switchgrass, with a cost of $0.003 (USD) per reaction for mesophyll protoplasts and $0.018 for axenic cell culture-derived protoplasts. Further, the efficiency of protoplast transformation was optimized threefold over previous methods, despite a fourfold reduction in DNA quantity. The methods developed in this work remove the cost barrier previously limiting high-throughput screening of genome-editing and gene silencing targets in switchgrass, paving the way for more efficient development of transgenic plants.

## Introduction

Over the last decade, associated with the rapid boom of “omics” technologies, there has been an increasing trend in the development of protoplast systems, for numerous plant species, for rapid gene screens and reverse genetics. Recently, protoplast isolation and transfection systems have been developed/improved for maize (*Zea mays*) (Cao et al. 2014), carrot (*Daucus carota*) (Maćkowska et al. 2014), poplar (*Populus euphratica*) (Guo et al. 2015), grape (*Vitis vinifera*) (Wang et al. 2015), oil palm (*Elaeis guineensis*) (Masani et al. 2014), lettuce (*Lactuca sativa*) (Sasamoto and Ashihara 2014), and mustard (*Brassica juncea*) (Uddin et al. 2015), just to name a few. The reemergence of protoplast systems is directly related to their utility in the analysis of protein subcellular localization (Chen et al. 2015; Nieves-Cordones et al. 2014), protein–protein interactions (Fujikawa et al. 2014; Li et al. 2015), transcriptional regulatory networks (Nakashima et al. 2014; Pruneda-Paz et al. 2014), signal transduction pathways (Cao et al. 2014), and rapid analysis of gene expression (Yoo et al. 2007). With the advent of genome-editing and gene silencing technologies, protoplast systems have found further utility due to the ease in screening the efficiency of numerous targets, e.g., dsRNA (Cao et al. 2014), siRNA (Bart et al. 2006), miRNA (Martinho et al. 2015), or gRNA (Xing et al. 2014) prior to the development of transgenic plants. With the renewed interest in protoplasts, significant progress has been made into the regeneration of protoplasts into whole plants, which further allows for the establishment of transgenic plants without the need for *Agrobacterium*-mediated transformation. For crop species, this is a crucial advantage, as transgenic plants that have been transformed by non-pathogen-related methods are not as heavily regulated. Despite these advantages, the widespread use of protoplasts is often hampered by the high cost of cell wall degrading enzymes, the large quantity of DNA required for transfection, the need for a constant source of tissue (leaves or roots) for isolation, and regeneration and fertility of regenerated plants, particularly in monocots. As an example of an important lignocellulosic bioenergy feedstock that could significantly benefit from a protoplast screening system, switchgrass (*Panicum virgatum* L.) was chosen for further study.

Previous research has demonstrated the economic viability of switchgrass as both an agricultural and biofuel crop (McLaughlin and Kszos 2005). Unfortunately, a major economic barrier to the broad use of switchgrass as a lignocellulosic feedstock is the recalcitrance of cell walls to digestion. In order to reduce recalcitrance, numerous studies have focused on the generation of transgenic plants with altered lignin and cell wall bound phenolics (Fu et al. 2011; Ragauskas et al. 2014; Shen et al. 2012, 2013). In addition, since switchgrass is a non-model crop, it has been necessary to identify promoters that can effectively regulate the expression of transgenes in switchgrass (Mann et al. 2011, 2012b). While some success has been attained in the generation of transgenic switchgrass with altered cell wall architecture, the current path from identification of target genes and promoters, through callus transformation, followed by phenotypic characterization in the greenhouse is extremely laborious and slow (Burris et al. 2009; Li and Qu 2011). While previous research has attempted to utilize switchgrass protoplasts for transient screening, the procedure was cost prohibitive, slow, and not very efficient (Mazarei et al. 2008). Considering the importance for rapid screening of promoter efficiency, genome-editing and silencing targets, and gene expression in switchgrass, the development of a rapid, low-cost protoplast isolation and transformation system was the primary objective of this work.

## Materials and methods

### Plant material

**Fig. 1:**
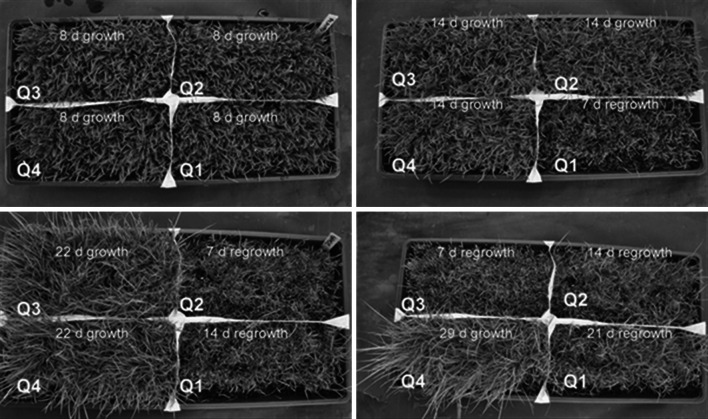
Schematic of switchgrass “lawns” demonstrating stage of growth of leaf tissue when harvested from each quadrant (*Q1*, *Q2*, *Q3*, *Q4*) at 8, 14, 22, and 29 days after planting and regrowth at 7, 14, 21, and 28 days following complete cutting of tissue

*Panicum virgatum* cv. Alamo seeds were obtained from Bemert Seed (Muleshoe, Texas, USA). For initial optimization, Alamo seeds were planted at an approximate density of 20 mg/cm^2^ in Fafard^®^ 3B soil mix (Sun Gro Horticulture, Agawam, Massachusetts, USA), and grown with a 16 h light, 4 h dark cycle at 22 °C to generate lawns of switchgrass plants in flats. For initial harvests, the plants were grown for 2 weeks, and then the leaves were cut with a scalpel to approximately 1.5 cm above the soil and used for protoplast isolation (see Fig. 1). For time-course experiments, each flat was divided into four quadrants in which tissue was harvested from each quadrant at 8, 14, 22, and 29 days after planting (Fig. 1). Regrowth was assessed 7, 14, 21, and 28 days following initial harvest.

*Panicum virgatum* cv. Alamo genotype ST1 cell suspension cultures were established from node culture as described previously (Alexandrova et al. 1996) and maintained in KM8 medium (Kao and Michayluk modified basal medium, Phytotechnology Laboratories, Overland Park, Kansas, USA) with the addition of 20 % sucrose, 10 % glucose, 0.025 % fructose, 0.025 % sorbitol, 0.025 % mannitol, 0.2 mg/L zeatin, 1 mg/L NAA, 0.1 mg/L 2,4-d (Kao and Michayluk 1975). Suspension cultures were incubated in the dark at 30 °C on a rotary shaker at 105 rpm. Liquid cell suspension cultures were subcultured every 5–7 days and callus cultures were subcultured monthly. Five days after subculture, ST1 cell suspensions were used to produce protoplasts.

### Protoplast isolation

Isolation of protoplasts from leaf tissue was adopted from the procedure described for *Arabidopsis thaliana* (Sheen 2001) with several modifications. Leaf protoplasts were isolated from mesophyll tissue in a buffer solution (0.6 M mannitol, 10 mM MES, 1 mM CaCl_2_, 5 mM 2-mercaptoethanol, and 0.1 % BSA, pH 5) containing food-grade enzymes at the manufacturer’s suggested concentrations (Rohament CL 1320 ECU, Rohapect 10L 840 ADJU, and Rohapect UF 0.0065 ADJU) (AB Enzymes, Darmstadt, Germany) and filtered through a 0.22 µm syringe filter (Millipore Express PES Membrane, Merk Millipore Ltd, Tullagreen, Carrigtwohill Co. Cork, Ireland). Leaf tissue was harvested from each quadrant at 8, 14, 22, and 29 days after planting (Fig. 1), cut into 2 mm strips in a Petri dish and weighed. Additionally, regrowth was assessed at 7, 14, 21, and 28 days following the initial harvest to determine whether the switchgrass lawn system could be used repeatedly over time without a decrease in the protoplast yield. Cut leaf tissue was added to the enzyme buffer solution (ca. 200 mg tissue/10 mL solution) and incubated with shaking at 80 rpm for 30 min to 24 h, at 28, 37, or 55 °C (maximum optimal temperature of food-grade enzymes was 60 °C) with or without protection from ambient light.

Following incubation, tissue and buffer mixture was filtered through a 40 µm filter (Fisherbrand, Fisher Scientific, Hampton, New Hampshire, USA). Five milliliters of W5 solution (154 mM NaCl, 125 mM CaCl_2_, 5 mM KCl, 2 mM MES, pH 5.7) was then passed through the same filter to dilute the enzyme solution and maximize protoplast collection. Protoplasts were collected and the enzyme solution was removed using centrifugation at 150×*g*, 22 °C for 10 min. Protoplasts were then resuspended in W5 solution, enumerated, and viability was assessed using propidium iodide (PI) staining (working solution: 1 mg/50 mL, Sigma–Aldrich, St. Louis, Missouri, USA). Protoplasts were placed on ice following isolation and prior to transfection.

Protoplasts were obtained from cell suspension cultures using similar methods as those for leaf mesophyll protoplasts. Twenty milliliters of a 5 or 8-day-old ST1 suspension culture was removed from a 200 mL culture and cells were allowed to settle for approximately 15 min. Most of the medium was removed from the cell suspension and approximately 10 % of the initial volume remained as cells. Twenty milliliters of buffer solution containing food-grade enzymes (Rohament CL 7920 ECU, Rohapect 10L 5040 ADJU, and Rohapect UF 0.039 ADJU) (AB Enzymes, Darmstadt, Germany) was added to the remaining cells (ca. 2 mL cells/20 mL solution) and incubated for 2 h at 30 °C. Following incubation, cells and buffer mixture were filtered through a 40 µm filter (Fisherbrand, Fisher Scientific, Hampton, New Hampshire, USA). Twenty milliliters of W5 solution was then added to the tube containing cells, mixed by inverting and passed through the same filter to dilute the enzyme solution and maximize protoplast collection. Protoplasts were collected and the enzyme solution was removed using centrifugation at 150×*g*, 4 °C for 10 min. Protoplasts were then resuspended in W5 solution, enumerated, and viability was assessed using propidium iodide (PI) staining (working solution: 1 mg/50 mL). Protoplasts were placed on ice following isolation and prior to transfection.

### Plasmid

The pANIC10A plasmid containing the *pporRFP* orange fluorescent reporter gene (OFP) under the control of the PvUbi1+3 switchgrass constitutive promoter was used in this study (Mann et al. 2011). To create a plasmid that could be readily isolated from standard *Escherichia coli*, the *mGFP5*-*ER* gene was inserted in reverse orientation using Gateway^®^ cloning, to remove the ccdB cassette, to generate the 16 kb pANIC10A GFPuv stuffer plasmid which was used for all transfection experiments. This plasmid was propagated in *E. coli* and purified using a ZymoPURE Giga Prep kit (Zymo Research, Irvine, CA).

### PEG-mediated transfection

PEG-mediated DNA transfection was performed as previously described (Sheen 2001) with modifications. Protoplasts were resuspended in MMg (0.4 M mannitol, 25–150 mM MgCl_2_, 4 mM MES (pH 5.7)) at a concentration of 1 × 10^6^ protoplasts/mL (leaf) or 2 × 10^5^ protoplasts/mL (cell suspension). Plasmid DNA (0–40 µg) was mixed with 200 µL of protoplasts (approximately 2 × 10^5^ protoplasts for mesophyll and 4 × 10^4^ protoplasts for cell suspension). Approximately 0–50 % PEG solution (0.6 M mannitol, 100 mM CaCl_2_, 0–50 % PEG 4000 (Sigma–Aldrich, St. Louis, Missouri, USA)) was added to the protoplasts to a final PEG concentration of approximately 0–25 %. After a 20 min incubation at room temperature, protoplasts were washed twice with approximately 1–4 mL of W5 and collected by centrifugation at 100×*g* for 5 min. Protoplasts were resuspended in 1 mL WI (0.6 M Mannitol, 4 mM KCl, 4 mM MES, pH 5.7), transferred to 12-well Falcon culture plates (Corning Incorporated, Corning, New York, USA) and incubated at 28 °C in the dark for 15–20 h. Microscopic evaluation of expression of the *pporRFP* reporter was conducted using an Olympus IX71 microscope with the Chroma 49004 CY3/TRITC filter set.

### Statistical analysis

A completely random experimental design was used for leaf protoplast optimization experiments, growth and regrowth experiments, and transformation experiments, with all containing at least three independent biological and technical replicates. Results were analyzed using mixed model ANOVAs (SAS 9.4, Cary, North Carolina, USA). Least significant differences (LSD) were used to determine significant differences among means when the ANOVA results were statistically significant (*p* < 0.05).

To calculate viable protoplasts per mg of starting tissue, the following equation was used:

$$\frac{\text{viable protoplasts}}{\text{mg tissue}} = \frac{{\left( {{\text{total}\, \# \text{ protoplasts} \times \% \,\text{viability}}} \right)}}{\text{mg starting tissue}}$$

The number of protoplasts expressing the OFP and the number of protoplasts not expressing OFP were counted using a hemocytometer. To ensure that a statistically significant distribution of protoplasts was counted on the hemocytometer, samples were collected from individual wells and centrifuged at 100×*g* prior to resuspension in a minimal volume ~100 µL. Using this strategy, an average of 78.9 protoplasts, across all transformation experiments, were counted on each hemocytometer grid. Transformation efficiency was calculated as:

$$\left( {\frac{{{\#\, \text{protoplasts expressing OFP}}}}{{{\text{total}\, \#\, \text{protoplasts}}}}} \right){\times 100 = \% \,\text{transformation efficiency}}$$

## Results

### Optimization of protoplast isolation using food-grade enzymes

**Fig. 2:**
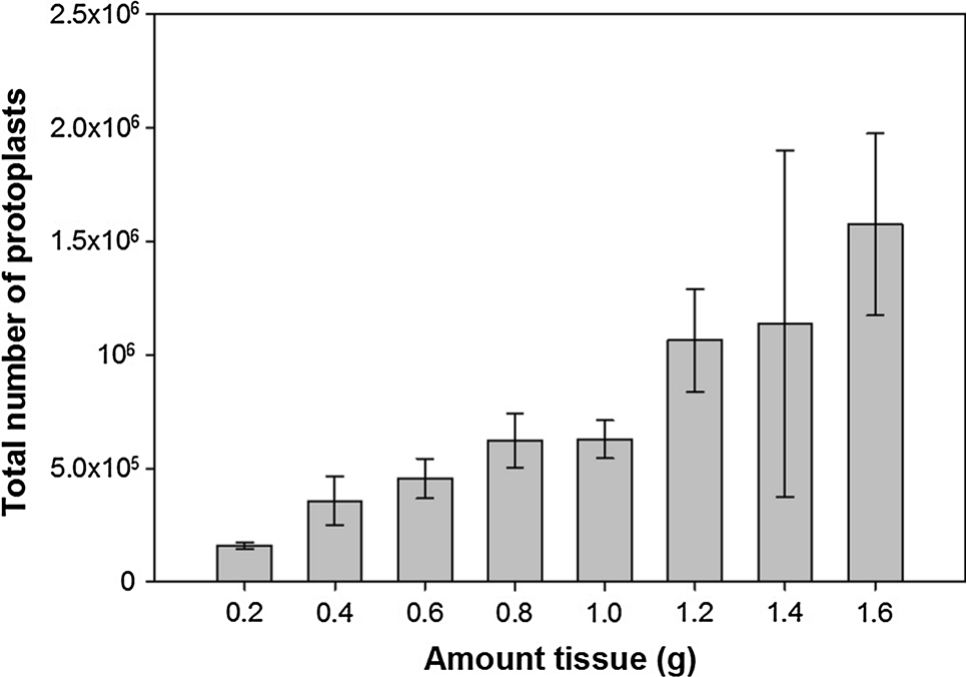
Total protoplast yield for varying amounts of leaf tissue. A concentration of 1320 ECU Rohament CL, 840 ADJU Rohapect 10L, and 0.0065 ADJU Rohapect UF, was able to digest >1.6 g of leaf tissue, without a change to the yield per milligram of tissue (*n* = 3). At the upper limit tested, ~1.6 × 10^6^ protoplasts could be generated from a single reaction. Protoplastation conditions: CL = 1320 ECU, 10L = 840 ADJU, UF = 0.0065 ADJU, 3 h digestion, temperature 37 °C, in the dark

**Fig. 3:**
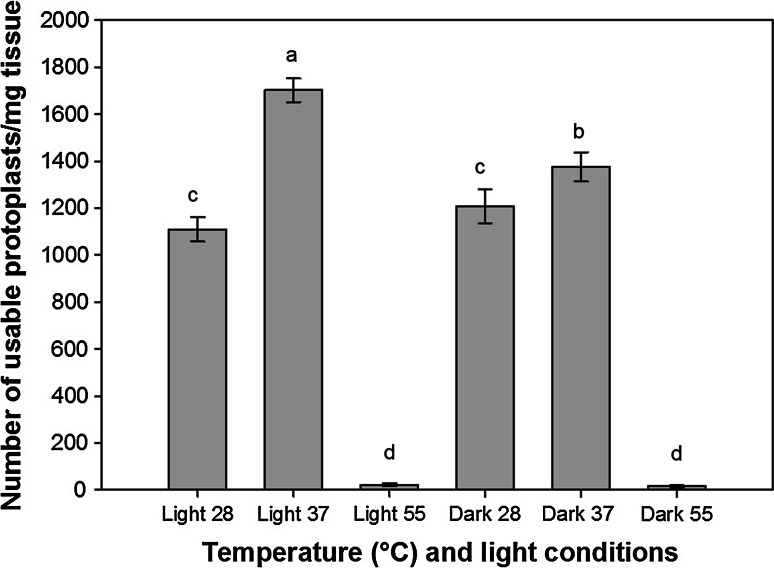
Temperature (28, 37 or 55 °C) and light conditions (light or dark) and the effect on viable protoplast recovery per mg starting tissue. Protoplastation conditions: CL = 1320 ECU, 10L = 840 ADJU, UF = 0.0065 ADJU, and 3 h digestion. *Error bars* represent standard error (*n* = 6). *Same letters above bars* indicate no significant difference according to the LSD test (*p* < 0.05)

**Fig. 4:**
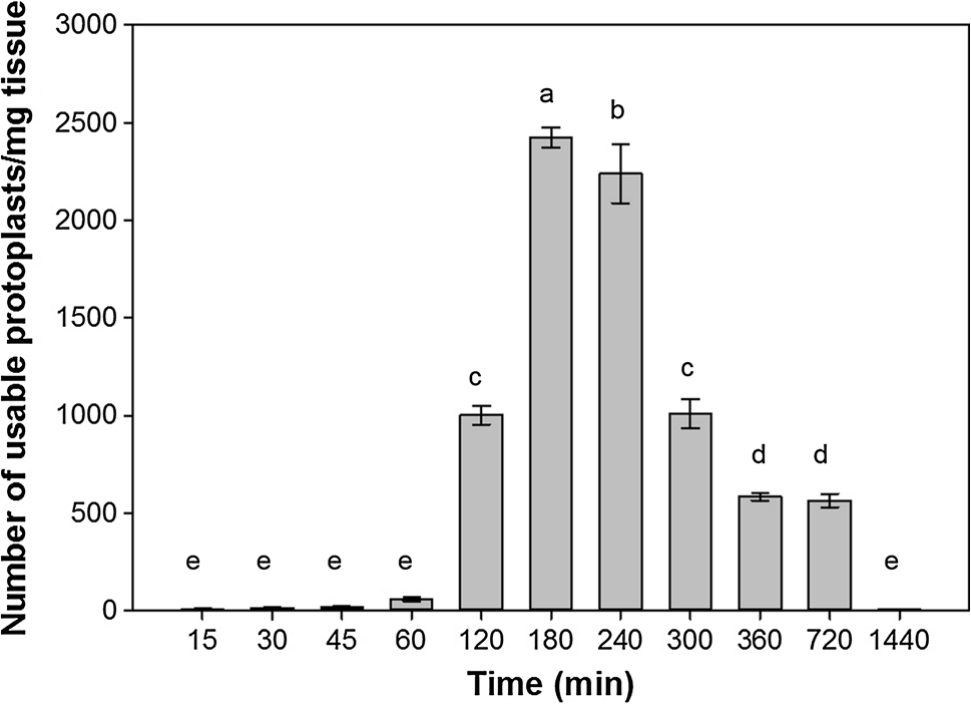
Time of incubation in enzyme mixture (min) and its effect on the number of viable protoplasts recovered per mg tissue. Protoplastation conditions: CL = 1320 ECU, 10L = 840 ADJU, UF = 0.0065 ADJU, temperature 37 °C, in light. *Bars* represent standard error (*n* = 6). Same letter above bar indicates no significant difference (*p* < 0.05) according to the LSD test

Recent research has demonstrated that the food-grade cell wall degrading enzymes Rohament CL, Rohament PL, and Rohapect UF may provide a low-cost alternative to lab-grade enzymes for protoplast isolation (Buntru et al. 2014, 2015). To test this hypothesis, isolation of protoplasts from switchgrass leaf tissue was tested using Rohament CL, Rohapect 10L, and Rohapect UF. At concentrations of 1320 ECU (Rohment CL), 840 ADJU (Rohapect 10L), and 0.0065 ADJU (Rohapect UF), >1.6 g of 2-week old leaf tissue could be digested without a loss in the protoplast yield per mg of tissue (Fig. 2). Based on this data, a trend line was fit to the dataset (*R*^2^ = 0.94) to obtain the protoplastation efficiency of 8.4 × 10^5^ protoplasts per gram of tissue. In order to optimize the method of protoplast isolation using these enzymes, the temperature of the digestion was analyzed, along with digestion in either light or dark conditions (Fig. 3). It was determined that digestion at 37 °C was optimal for both light and dark conditions (*p* < 0.05), with a maximum protoplast yield of 1702 ± 50 viable protoplasts per mg of tissue in the light and 1375 ± 62 viable protoplasts per mg of tissue in the dark. Surprisingly, at 37 °C, there was a significant increase in protoplast yield with incubation in the light, compared to the dark conditions (*p* < 0.05). At both 28 and 55 °C, there was no significant difference between the light and dark treatments; however, incubation at 55 °C resulted in a decrease in viability leading to less than 200 viable protoplasts per mg of tissue, a > 9-fold decrease compared to the 37 °C treatment (Fig. 3). To further optimize the procedure, the duration of digestion was tested over 24 h to identify the time required to maximize the yield of viable protoplasts. From these results, it was determined that the maximum number of viable protoplasts per mg of tissue (2424 ± 56) was recovered after digestion for 180 min (*p* < 0.05) (Fig. 4). While there was a slight reduction of 7.7 % in the number of viable protoplasts per mg of tissue at 240 min, digestion at >240 min and <180 min resulted in less than half of the maximum yield (Fig. 4). It should be noted that since the yield has been converted to the number of viable protoplasts per mg of tissue, at <180 min there are less total protoplasts due to incomplete digestion, whereas at >240 min there is a decrease in viability but not total protoplasts. Based on the results from these experiments, it was determined that the optimum protoplast isolation procedure with Rohament CL, Rohapect 10L, and Rohapect UF for switchgrass was a 180 min digestion at 37 °C in the light.

### Analysis of a renewable source for switchgrass leaf tissue

**Fig. 5:**
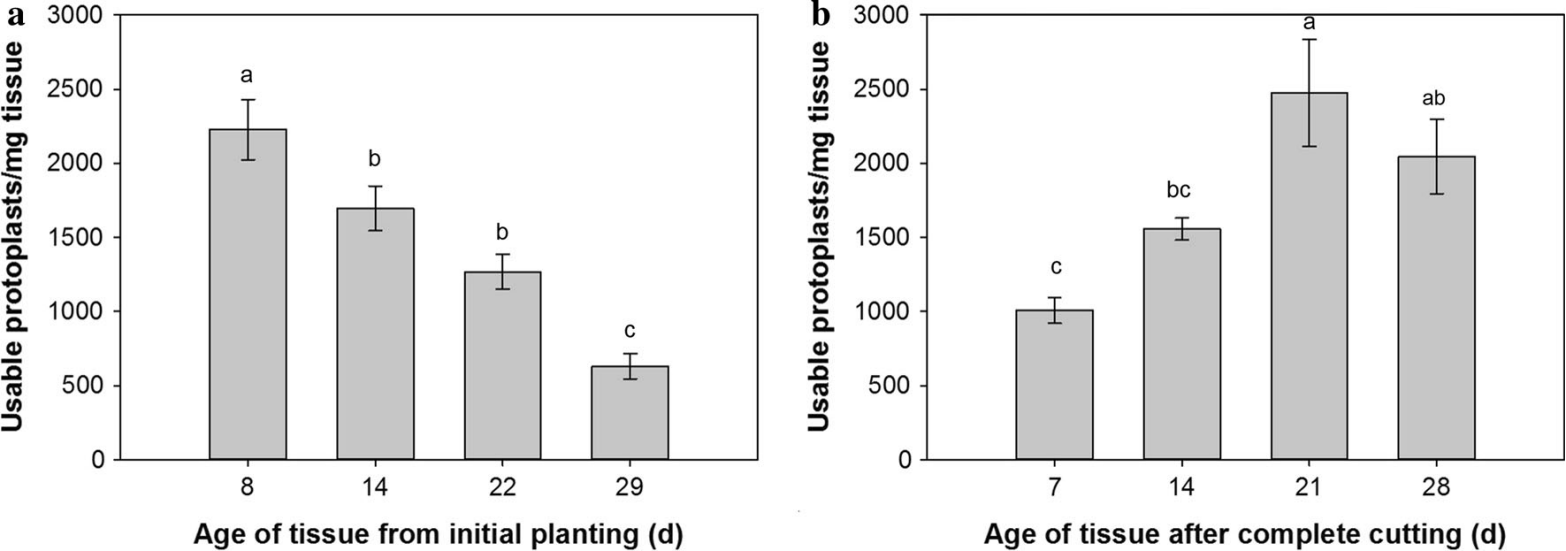
Effect of tissue age on viable protoplast recovery per mg of starting tissue. **a** Age (in days) at switchgrass tissue harvest and its effect on the viable protoplast recovery per mg of starting tissue. **b** Age (in days) at switchgrass tissue harvest after complete cutting (regrowth) and its effect on the viable protoplast recovery per mg starting tissue. Protoplastation conditions: CL = 1320 ECU, 10L = 840 ADJU, UF = 0.0065 ADJU, 3 h digestion, temperature 37 °C, in light. *Error bars* represent standard error (*n* = 9). *Same letters above bars* indicate no significant difference (*p* < 0.05) according to the LSD test

The need for a renewable supply of tissue with a limited footprint, i.e., without the need for greenhouse space, was a consideration of this work. As such, switchgrass “lawns” were established for the generation of leaf tissue for protoplast isolation (Fig. 1). Harvesting of tissue at weekly intervals showed a gradual decrease in the protoplast yield over a 4-week period, with a maximum (2230 ± 204 viable protoplasts per mg of tissue) at 8 days after initial planting (Fig. 5a). After 14–22 days, approximately a 33 % reduction in yield was observed, with a reduction of 72 % after 29 days. After identifying the ideal time for first harvest, to test the sustainability of the lawn system, the yield of protoplasts from re-growth after the initial harvest was also examined. After re-growth for 14 days, the yield of protoplasts was similar to the initial harvest at 14–22 days (1560 ± 758 viable protoplasts per mg of tissue) (Fig. 5b). While the maximum protoplast yield from the re-growth was achieved at 21 days (2480 ± 363 viable protoplasts per mg of tissue), there was no significant difference in yield from 7 days (Fig. 5b). The lack of significance in the yield for the re-growth data is most likely due to difficulty in manually cutting at the same level in the initial harvest. However, even at the minimal yield attained in the re-growth study (1010 ± 87 viable protoplasts per mg of tissue), the level was not significantly different from the initial 22 day harvest (1270 ± 117 viable protoplasts per mg of tissue) (*p* = 0.09). Based on this data, the same lawn can be used for multiple harvests, which reduces the need for continuous planting. Further, continued experiments have determined that repeated cutting/re-growth did not decrease the yield of protoplasts for up to four cycles, extending the sustainability of a single planting to ~3.5 months.

### Optimization of switchgrass protoplast transformation

**Fig. 6:**
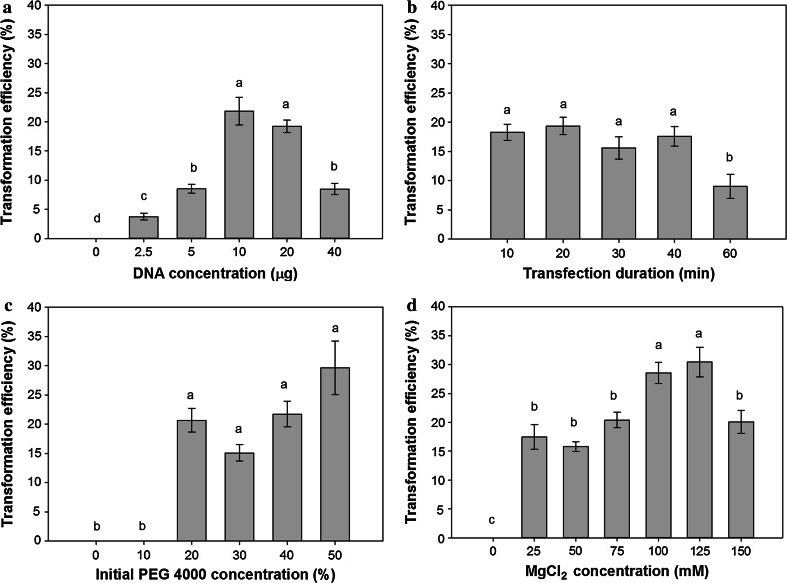
Optimization of transformation for switchgrass protoplasts. **a** Effect of DNA concentration on transformation efficiency. **b** Effect of duration of transfection (min) on transformation efficiency. **c** Effect of PEG 4000 concentration on transformation efficiency. **d** Effect of MgCl_2_ concentration on transformation efficiency. *Error bars* represent standard error (*n* = 6). *Same letters above bars* indicate no significant difference (*p* < 0.05) according to the LSD test

Optimization of a transformation protocol for switchgrass mesophyll protoplasts was conducted to study the effects of plasmid concentration, transfection duration, MgCl_2_ concentration, and PEG 4000 concentration on the transformation efficiency of switchgrass protoplasts. The first variable that was optimized was the amount of pANIC10A GFPuv stuffer plasmid (0–40 µg) required for transformation. The highest transformation efficiency (21.8 ± 2.3 %) was achieved with a DNA concentration of 10 µg, although there was no significant difference between 10 and 20 µg of DNA (*p* = 0.34) (Fig. 6a). Surprisingly, at a concentration of 40 µg, transfection efficiency decreased 2.4 times and was not significantly different from the reactions with 5 µg of DNA (*p* = 0.98) (Fig. 6a). The second variable that was optimized was the duration of the transfection procedure. Based on the results from these experiments, there was no significant difference in the transformation efficiency from 10 to 40 min (*p* > 0.05); however, after 60 min, the transformation efficiency was reduced by 1.8 times compared to the shorter duration reactions (*p* = 0.006) (Fig. 6b). Similar to the results for the reaction duration, at initial PEG 4000 concentrations of 20–50 %, there was no significant difference in the transformation efficiency (21.8 ± 8.4 %, *p* > 0.05) (Fig. 6c). However, below a concentration of 20 % PEG 4000 no transformation was observed, identifying this concentration as the minimal PEG 4000 required to achieve transformation of switchgrass protoplasts (Fig. 6c). While duration of the reaction and PEG 4000 concentration had little effect on increasing the transformation efficiency, a significant increase was observed when the MgCl_2_ concentration was increased from 25 to 100–125 mM (*p* < 0.05) (Fig. 6d). A maximum transformation efficiency (30.4 ± 2.5 %) was observed at 125 mM and was 1.65 times greater than MgCl_2_ concentrations ranging from 25 to 75 and 150 mM (18.4 ± 4.2 %) (Fig. 6d). Based on the data obtained for optimization of transformation in switchgrass protoplasts, the optimal method was found to be incubation of 10 µg of plasmid for 10–40 min with an initial PEG 4000 concentration of 20–50 % and a MgCl_2_ concentration of 100–125 mM. Using this method, a maximum transformation efficiency of 30.4 % was attained from switchgrass mesophyll protoplasts.

### Isolation and transformation of cell culture-derived protoplasts

**Fig. 7:**
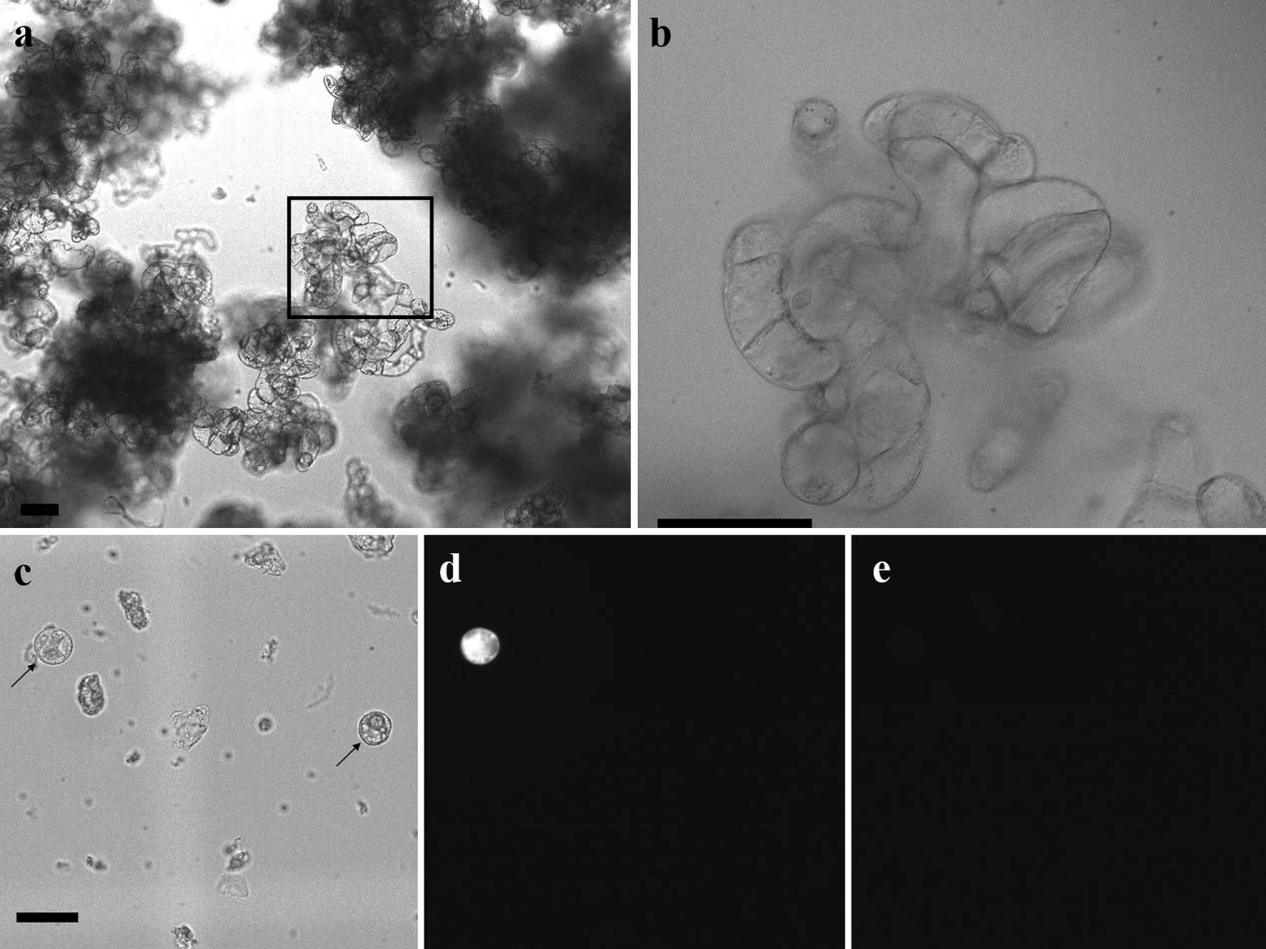
ST1 cell culture and protoplasts isolated from culture. **a** Low-magnification (10X) image of population of 8-day-old ST1 cell culture grown in KM8. *Scale bar* is 10 µm. **b** High-magnification (40X) image of boxed portion of cell culture in **a**. *Scale bar* is 100 µm. **c** Expression of OFP reporter in protoplasts isolated from ST1 cell suspension culture 18 h following transfection with 10 µg pANIC10A GFPuv stuffer plasmid DNA was visualized using a tdTomato filter set: 535/30 nm excitation and 600/50 nm band pass emission and GFP filter set: 535/30 nm excitation and 600/50 nm band pass emission. The exposure time was 20 ms under white light (**c**), tdTomato filter (**d**) and GFP filter (**e**). Protoplasts shown with *arrows* in **c**. *Scale bar* is 10 µm

Since cell suspensions have proven to provide a constant source of sterile, rapidly growing cells, capable of generating protoplasts in other systems (Doelling and Pikaard 1993; Wang et al. 2015), a switchgrass cell culture system for generation of protoplasts was developed. Switchgrass cell suspension cultures were established from callus of the clonal Alamo ST1 cultivar following previously established methods (Gupta and Conger 1999) with several variations. Briefly, after initiation of callus on LP9 media (Burris et al. 2009), callus was transferred to liquid KM8 media and axenic cultures were allowed to establish for a period of 1 month, followed by subculturing every 5–7 days thereafter. ST1 cell suspension cultures were comprised of large aggregated cells (Fig. 7a, b), and displayed rapid growth, ideal for protoplast harvesting. After establishing the cultures, isolation of protoplasts from the cell suspensions were attempted using the optimized method for leaf mesophyll protoplast isolation described above. Unfortunately, the mesophyll protocol failed to release protoplasts from the cell culture, leaving predominately intact cells. Therefore, the enzyme concentrations were increased sixfold, similar to previous work on cell culture protoplasts (Mazarei et al. 2011), to 7920 ECU for Rohament CL, 0.039 ADJU for Rohapect UF, and 5040 ADJU for Rohapect 10L. Results from digestion with the elevated enzyme concentrations found that 3.14 × 10^5^ ± 3.35 × 10^4^ viable protoplasts could be harvested from a packed cell volume (PCV) of 3 mL, with no significant difference between isolation at 28 and 37 °C (*p* = 0.94). The protoplastation efficiency of the suspension cultures was 9.6 × 10^5^ protoplasts per gram of cells, as determined by the weight of a 3 mL PCV after filtration through a 3 µm mesh to remove excess water. Unlike the difference in protoplast isolation methods between the mesophyll and cell culture-derived protoplasts, the optimized transfection protocol was significantly more efficient with the cell culture-derived protoplasts isolated at 28 °C, with an efficiency of 46.4 ± 3.3 % (*p* < 0.05) (Fig. 7c–e). Surprisingly, there was a significant reduction (*p* < 0.05) in the transformation efficiency of cell culture-derived protoplasts (25.4 ± 3.3 %) isolated at 37 °C.

## Discussion

Traditionally, protoplast isolation from plants and fungi use highly purified lab-grade cell wall-digesting enzymes, with many protocols specifying a vendor to ensure success of a procedure (Yoo et al. 2007). Often lab-grade enzymes for protoplast isolation are very costly with the enzyme cost often prohibitive to high-throughput research. For example, based on the previous methodology for switchgrass protoplast isolation (Mazarei et al. 2008) from approximately 130 mg of leaf tissue, the cost per reaction was $11.59 for the enzymes alone. Considering that each reaction generated ~8×10^5^ protoplasts, a maximum of four transfection experiments (typically 2 × 10^5^ protoplasts are used for transformation) could be conducted per reaction, with a cost per transfection of $2.89 for the enzymes alone. Recent research has demonstrated that the use of the low-cost food-grade enzymes, Rohament CL, Rohament PL, and Rohapect UF provides a significant reduction to the cost of protoplast isolation for the Bright Yellow 2 (BY-2) tobacco cell culture line (Buntru et al. 2014, 2015). In this system, Rohament CL provides the cellulase activity, Rohament PL provides the pectinase activity, and Rohapect UF supplements the other enzymes with specialized pectinases and arabinases (Buntru et al. 2014). Since food-grade enzymes have successfully been used to isolate protoplasts from tobacco, with significantly reduced costs, similar food-grade enzymes (Rohament CL, Rohapect 10L, and Rohapect UF) were tested in this work for their ability to release protoplasts from switchgrass leaves. Using these enzymes, it was possible to reduce the cost of mesophyll protoplast isolation to <$0.01 per reaction (based on current pricing from AB Enzymes), a greater than 1000-fold decrease compared to previous methods. Further, the concentration of enzymes used were able to digest >1.6 g of tissue (Fig. 2), releasing ~1.5 × 10^6^ protoplasts per reaction, nearly doubling the yield of mesophyll protoplasts compared to previous methods. The development of a low-cost protoplast isolation system represents an important step in realizing high-throughput screening of transgene expression and promoters in switchgrass; however, to realize this goal, a reliable transformation system is required.

While callus-based *Agrobacterium tumefaciens*-mediated transformation is standard for plant transformation, including switchgrass (Burris et al. 2009; Li and Qu 2011), this method has many disadvantages, including regulatory restrictions (Garrett 1987; Jaffe 2004), limited control of insertion rates resulting in variation in transgene insertion and expression (Hobbs et al. 1993), and potential recovery of chimeric plants (Dominguez et al. 2004). Specifically for switchgrass, *Agrobacterium*-based transformation efficiency is inconsistent and can depend upon genotype, callus type, and callus age (Burris et al. 2009; Li and Qu 2011). Additionally, a high frequency of false positives, up to 30 %, has been reported from callus transformation of switchgrass (Ogawa et al. 2014; Somleva et al. 2002). Since protoplasts are devoid of cell walls, a necessary attachment point for *Agrobacterium*, protoplasts cannot be transformed via *Agrobacterium*. However, the lack of a cell wall opens the door for non-*Agrobacterium*-based transformation protocols, which are routinely used in mammalian systems. Previous studies have used electroporation- (Fromm et al. 1985; Negrutiu et al. 1987), polyethylene glycol (PEG)- (Armstrong et al. 1990; Negrutiu et al. 1987), nanoparticle- (Silva et al. 2010), and lipofection- (Felgner et al. 1987) mediated transformation of plant protoplasts with varying success. Specifically, previous work on switchgrass protoplasts used PEG-mediated transformation with 40 µg of a 5.6 kb plasmid, and achieved very low efficiency transformation (Mazarei et al. 2008). Similar to the high enzyme cost, 40 µg of plasmid DNA per reaction represents a significant hurdle to high-throughput screening of protoplasts, and will discourage many labs from utilizing this protoplast system. Therefore, optimization of a transformation protocol for switchgrass mesophyll protoplasts was conducted to study the effects of plasmid concentration, MgCl_2_ concentration, PEG 4000 concentration, and transfection duration on transformation efficiency.

As a “worst-case” scenario the 16 kb pANIC10A GFPuv stuffer plasmid was chosen for evaluation of transformation efficiency. A large plasmid would likely be necessary for CRISPR genome-editing studies, or more complex multi-gene expression studies. Typically, smaller plasmids in the 5 kb range are used for PEG-mediated transformation (Mazarei et al. 2008; Sheen 2001), which may bias the efficiency reported towards these simpler systems. Based on the results obtained from the optimization experiments, a fourfold reduction in the DNA content increased the switchgrass protoplast transfection efficiency by twofold, over the previous methodology (Mazarei et al. 2008). Compared to grape and maize protoplasts, the DNA content required for optimal transformation efficiency in switchgrass was two to tenfold lower, respectively (Cao et al. 2014; Wang et al. 2015). Previous research has noted that decreasing DNA titer often reduces labor and material costs, while potentially increasing efficiency of protoplast transformation (Armstrong et al. 1990; Damm et al. 1989; Maas and Werr 1989). Unlike the increased transformation efficiency observed with a reduction in DNA content, the concentration of PEG 4000 in the reaction mixture had little effect on the efficiency of transformation. Whereas in previous protoplast systems where lower transformation efficiencies have been observed when PEG 4000 surpasses 25 % (Masani et al. 2014), due to toxicity of PEG itself, no PEG toxicity was observed with switchgrass protoplasts even with the highest levels tested. Not surprisingly, the most significant increase in transformation efficiency was achieved by increasing the MgCl_2_ concentration from 15 to 100–125 mM. Previous studies have demonstrated that MgCl_2_ concentration contributes significantly to the efficiency of PEG-mediated transient gene expression in tobacco (Negrutiu et al. 1987), maize (Armstrong et al. 1990) and oil palm protoplasts (Masani et al. 2014). Through optimization of the transfection procedure, it was possible to increase protoplast transformation efficiency from 9.1 to 30.4 %, while also reducing the quantity of DNA by fourfold.

In addition to the differences in the transformation efficiency between the mesophyll and cell culture-derived protoplasts, several other considerations were made when analyzing transformed protoplasts from each source. First, the average fluorescent intensity of the cell culture-derived protoplasts was greater than the mesophyll protoplasts. Since quantitative data was not obtained for fluorescence, this observation was made by using the same exposure setting for analyzing transgenic protoplasts from each source. This increased intensity may be due to higher metabolic activity and more rapid growth in the cell culture protoplasts, or may also be due to the more consistent protoplast size. In general, protoplasts isolated from leaves had a wider size distribution than protoplasts isolated from the cell culture, which is not surprising due to the more consistent environment of a cell culture. Second, the mesophyll protoplasts had numerous chloroplasts present in the cell, while the cell culture protoplasts (grown in the dark) were devoid of chloroplasts. The presence of chloroplasts in isolated protoplasts was a factor in the choice of a fluorescent reporter, and led to the selection of *pporRFP*, which has an excitation maximum at 578 nm and emission maximum at 595 nm (Mann et al. 2012a). The use of *pporRFP* allowed selection of a filter set (Excitation 545/25x, Longpass 565, and Emission 605/70) that cut-off chlorophyll autofluorescence, while still allowing imaging of the marker. The combination of *pporRFP* with the filter set chosen for this work allowed imaging of transgenic protoplasts from both the cell cultures and leaf tissue, without any observable autofluorescence (Fig. 7c–e). It should also be noted that if mesophyll protoplasts were examined using a traditional Texas Red^®^ filter set, the chlorophyll autofluorescence dominated and prevented analysis of the *pporRFP* marker. Finally, as anticipated, transformed mesophyll protoplasts could only be screened for ~36 h before bacterial and fungal contamination dominated the cultures and killed the protoplasts. While antibiotics could be added to the protoplast isolation media to reduce contamination, this was not attempted in this work. Similarly, growth of aseptic seeds on agar in a sterile environment could be achieved, but would add additional costs and labor, and thus was not conducted in this study. Unlike the mesophyll protoplasts, the cell suspension-derived protoplasts could be maintained in soft agar cultures for up to 21 days (maximum duration tested) without contamination or a loss in expression of fluorescent marker. Despite the long duration of these cultures, no cell division or regeneration was observed; however, cytoplasmic streaming was evident throughout. Based on these comparisons, either system may function in rapid screening applications; however, for longer-term studies, the use of cell culture-derived protoplasts has a distinct advantage.

High efficiency transformation is essential for rapid screening, as typical reactions contain 2 × 10^5^ protoplasts, and the previous transformation efficiency (9.1 %) would generate 1.8 × 10^4^ OFP expressing protoplasts, below the level of detection of most plate readers. The increase in transfection efficiency demonstrated in this work (30.4 %) would result in 6.1 × 10^4^ OFP expressing protoplasts, within the range of standard plate readers. In addition, the reduction in DNA content to 10 µg will further reduce the cost of the entire procedure, and considering that a 16 kb plasmid was used for optimization, higher transformation efficiencies would be expected with smaller plasmids. Similarly, to achieve similar transformation efficiencies with an 8 kb plasmid would require half the DNA content as a 16 kb plasmid, as two times the number of individual plasmids would be present per reaction. The broader impact of a high-throughput protoplast screening system for switchgrass would be the ability to collect data at an earlier stage; therefore, screening out ineffective transgenes/promoters decreasing the number of plants to be recovered. For example, in a CRISPR study targeting recalcitrant genes, screening of gRNA targets in a protoplast system prior to the generation of transgenic plants would allow selection of targets with the highest efficiency of silencing. In this way, poor performing gRNA targets could be removed from the pool of candidates, generating a better chance of success in recovering the desired phenotype in greenhouse and field studies.

While the development of a low-cost mesophyll protoplast isolation system for switchgrass represents a significant improvement over current methodologies in both yield and cost, to obtain axenic protoplasts for long-term studies and potential regeneration, a switchgrass cell culture is necessary. Previous attempts at protoplast isolation from cell cultures in switchgrass were only successful with a single genotype, Alamo 2, and required four times the cellulase, two times the macerozyme, and the addition of driselase and pectolyase (Mazarei et al. 2011). The use of higher enzyme concentrations and the addition of other enzymes to the digestion increased the cost to >$60 per reaction, making the procedure cost prohibitive. Further, the cell cultures derived from Alamo 2 exhibited different morphologies (sandy, fine, and milky) from the same primary culture with only the milky culture yielding viable protoplasts (Mazarei et al. 2011). In order to develop a cell culture that was more feasible for large-scale protoplast isolation, in this work a cell suspension culture was established using the ST1 cultivar. Unlike the previous work, in which MS-maltose media was used to generate switchgrass callus, the callus used for initiation of the cell cultures was grown on LP9 media with sucrose as the sugar source. LP9 media has a decreased level of 2,4 dichlorophenoxyl-acetic acid (2,4 d; 5 mg L^−1^), increased proline (500 mg L^−1^), and no benzyladenine (BAP) or myo-inositol, which has been shown to be more effective for culturing switchgrass callus (Burris et al. 2009). The change in callus initiation and cultivation medium led to a more consistent type of culture, similar to the BY-2 tobacco cell culture (Fig. 7a, b) (Nagata et al. 1992). The fine, milky, and sandy types of culture observed for the Alamo 2 derived cultures were not observed in the ST1 suspension cultures established in this work, even after passage for over 6 months.

Unfortunately, application of the optimized mesophyll protoplast isolation procedure to the ST1 suspension cultures was not successful in isolation of the protoplasts. Considering that similar results were observed for Alamo 2 suspension cultures, the concentrations of Rohament CL, Rohapect 10L, and Rohapect UF were increased sixfold to match the cellulase concentrations used for digestion of previous switchgrass cell cultures. As indicated earlier, at this level, without the addition of driselase or pectolyase, it was possible to obtain 3–4 × 10^5^ protoplasts from a packed cell volume of 3 mL. The cost associated with the increased concentrations of the low-cost enzymes was minimal, with an overall cost of $0.018 per reaction. Considering the advantages of axenic switchgrass protoplasts, and the marginal increase in the cost of the reaction, the use of the ST1 switchgrass suspension culture provides an ideal method for rapid, bulk harvesting of switchgrass protoplasts for high-throughput studies.

While the protoplast isolation system developed in this work has utility in high-throughput screening applications, future research will be aimed at examining the potential to regenerate protoplasts isolated using this methodology. It is well established that monocot protoplast regeneration is difficult, with limited success in rice, wheat, and grasses (Harris et al. 1988; Kyozuka et al. 1987; Dalton 1988). Often nurse cultures or a complex series of different media is necessary to initiate regeneration, with the majority of regenerated plants being infertile. Specifically for switchgrass, protoplasts have not previously been regenerated, although suspension cultures have successfully been used to regenerate fertile plants (Gupta and Conger 1999). Of further concern would be impurities in the food-grade enzymes, not present in lab-grade enzymes that may interfere with the process of regeneration. However, if methods for regeneration of these axenic protoplasts could be developed, then it will be possible to extend the procedures developed in this work for the generation of transgenic plants without the need for *Agrobacterium*-mediated transformation. This would represent a fundamental shift in the generation of transgenic switchgrass, and increase the potential to overcome current limitation of recalcitrance in the cell walls of switchgrass.

### **Author contribution statement**

EMD and AGC conducted experiments and collected data for protoplast isolation, KPB and EMD conducted experiments for optimization of transformation, and AGC and KPB established and maintained the cell cultures. SCL, KPB, and CNS were responsible for data analysis and writing of the manuscript. All authors agree with the content of the manuscript.
